# Novel primary care treatment package for patients with medically unexplained symptoms: a cohort intervention study

**DOI:** 10.3399/bjgpopen17X101121

**Published:** 2017-10-04

**Authors:** Frank Röhricht, Ivan Zammit, Nina Papadopoulos

**Affiliations:** 1 Psychiatrist and Associate Medical Director, General Adult Psychiatry, East London NHS Foundation Trust, London, UK; 2 Honorary Professor, Centre for Psychoanalytic Studies, University of Essex, Colchester, UK; 3 Psychiatry Trainee Doctor, East London NHS Foundation Trust, London, UK; 4 Psychologist and Dance Movement Psychotherapist, East London NHS Foundation Trust, London, UK

**Keywords:** primary health care, medically unexplained symptoms, body oriented psychological therapy

## Abstract

**Background:**

Existing care models for patients with persistent medically unexplained symptoms (MUS) do not adequately address the needs of these patients. New and innovative intervention strategies are necessary to achieve better health and corresponding economic outcomes.

**Aim:**

To explore the feasibility of implementing a pragmatic care package that provides primary care treatment for patients with persistent MUS and to evaluate recruitment, retention, and acceptability as well as the potential impact on clinical outcomes and service utilisation.

**Design & setting:**

Prospective cohort intervention study involving a cluster of seven GP surgeries in Newham, East London, providing a 'One-Stop-Shop' primary care treatment service.

**Method:**

The care package included: identification, assessment, engagement, psychoeducation, and a choice of group interventions (mindfulness-based stress reduction [MBSR] and body-oriented psychological therapy [BOPT]). Baseline and follow-up data on somatic symptom levels (PHQ-15), health-related quality of life (SF-36, EQ-5D) and service utilisation was analysed.

**Results:**

In total, 145 patients were referred and assessed for eligibility, and 93 were included in the study. Participants engaged well with different components of the care package and gained significant improvements in somatic symptom levels with corresponding increases of quality-of-life ratings and a reduction in healthcare utilisation (GP contacts and referrals to specialist services) as well as associated healthcare costs.

**Conclusion:**

The primary care treatment package can be successfully implemented in primary care at a relatively low cost and easily adopted into routine care. The body-oriented approach is well accepted by clinicians and patients. Controlled trials should be conducted to test the efficacy of the treatment package.

## How this fits in

MUS are complex presentations that are common in primary care and pose a significant burden to patients, clinicians, and society due to a high level of unmet healthcare needs. GPs play an important role in assessment, engagement, and signposting for treatment. Take-up of psychological (talking) therapies among people with MUS through traditional referral systems and response rates for talking therapies are known to be low. This feasibility study suggests that a novel package, embedded in primary care, can be easily implemented and provide an alternative, and potentially cost-effective and clinically relevant, pathway.

## Introduction

Patients with MUS (also labelled bodily distress syndrome in newer classification systems),^1,2^ report experiencing physical symptoms that cannot be explained adequately or sufficiently by organic pathology, but which cause distress and functional impairment. As the causes remain unknown, the medical practitioner can diagnose MUS only by exclusion; however, research has demonstrated a high level of diagnostic accuracy.^3^ The fundamental assumption is that the complaints are not exclusively physical or mental in nature and origin, but complex presentations that cannot be assigned to a single causative factor.^4^

Persistent MUS (lasting >3 months) is highly prevalent and costly to patients, providers, and society; despite frequent presentation at primary and secondary care services, patients with MUS often have unmet health needs as a result of their health beliefs, incorrect diagnosis, and, consequently, ineffective treatment.^5–7^ Previous research emphasised the importance of GPs’ ability to provide generic interventions, such as positive communication and reassurance, as well as specific advice;^8^ in addition, studies found that enhanced primary care with input from specialists works best,^9^ and that an attitude of diagnostic openness is important.^10^ Flexible treatments with evidence supporting their efficacy include:

- reattribution therapy;
- progressive muscle relaxation; and
- cognitive behavioural therapy.^11^

A systematic analysis of non-pharmacological treatments, however, concluded that the effect sizes in trials evaluating psychological therapies for MUS have been low and that:

*'... compared with enhanced or structured care, psychological therapies generally were not more effective for most of the outcomes.'* ^12^

In addition, it has frequently been reported in trials that engaging significant numbers of patients in psychological care has been difficult.^12^

Individuals with MUS require a seamless care pathway and treatment package that is both flexible and multifaceted to meet their individual needs and to foster collaborative relationships.^13–14^ Existing standard models have not met those needs sufficiently to achieve the desired health outcomes among this group. It has been suggested that a significant improvement in therapeutic engagement and symptom reduction can be achieved while offering a symptom-focused integrative and flexible approach that includes experiential body-oriented psychological interventions.^15–17^

The cohort study presented here evaluated the feasibility and the potential clinical–cost implications of a novel care package that provides seamless primary care offering identification, assessment, engagement, and body-oriented interventions to patients with MUS. It aimed to address the following specific research questions:

- Is it feasible to implement the new care package within a primary care setting (a cluster of GP surgeries in East London) in terms of the practicalities relating to patient and health professional engagement?
- To what extent do patients' symptoms (psychological and physical complaints/distress) and their corresponding subjective quality of life change while receiving the care package?
- What is the potential impact of the care package on service utilisation and associated healthcare costs?

## Method

Training sessions were conducted for surgery staff on the specific characteristics and requirements of MUS care, engagement strategies for people with MUS, and to introduce the clinical algorithm for identifying potentially suitable participants.

The care package facilitated an ongoing clinical dialogue between GPs and practitioners who delivered the interventions.

### Study participants

Potentially eligible patients were identified as follows:

- from the primary care electronic database: computerised searches of clinical records were undertaken, using Read Codes on somatoform disorder and MUS, and specific conditions such as fibromyalgia;
- clinically by their GPs, according to the clinical algorithm; and
- through self-referrals.

Following identification, all potentially suitable patients were contacted by a member of the care team by telephone, letter, or face to face in a conversation during a routine consultation. Verbal consent for referral to the study was obtained and, once referred, a research assistant arranged for a baseline assessment to be done and written consent-giving procedures to be carried out.

Eligible patients comprised adults aged 18–75 years, who met the following criteria:

- persistent (≥6 months) bodily complaints without sufficient explanatory organ pathology (nature and degree according to GP judgement; using a screening algorithm: pain in different locations, non-specific complaints affecting multiple organ systems, repeated complaints of fatigue or exhaustion, symptoms occur in context of stressful lifestyle or stressful life events); at least mild somatic symptom severity on the 15-item Patient Health Questionnaire (PHQ-15), represented by a cut-off score of >5;^18^ and/or
- all patients with a diagnosis of undifferentiated somatoform disorder, classified as *The Diagnostic and Statistical Manual of Mental Disorders, fourth edition, *(DSM−IV) 300.81/82 and/or the 10th revision of the *International Statistical Classification of Diseases and Related Health Problems* (ICD−10) F45.

Patients were not eligible for inclusion if they had:

- somatisation symptoms attributable to identified physical disease (nature and degree);
- a primary diagnosis of anxiety or a depressive disorder, psychosis, substance misuse, psycho-organic disorder, and were considered to be actively suicidal; and
- insufficient language skills or an inability to complete the questionnaires.

### Data collection

Somatic complaints or symptom scores and health-related quality-of-life ratings were collected at baseline and at 4–6 months using:

- PHQ-15: scores of 5, 10, and 15, were the cut-off points for low, medium, high somatic symptom severity;^18^
- the physical and mental components of the 36-item Short Form Survey (SF-36): higher scores indicated a better health status;^19^ and
- Part 2 (Visual Analogue Scale, 0–100) of EQ-5D: higher scores indicated a better quality of life.^20^

Group interventions were assessed immediately after patient participation, using the Client's Assessment of Treatment Scale (CAT);^21^ this included quotations from patients as recorded in CAT questionnaires. Two authors read the free-text statements and determined the most important themes regarding therapeutic benefits.

All patients in the study were offered the opportunity to be seen in person for a follow-up assessment and interview by the research assistant; those who did not attend for follow-up received questionnaires (PHQ-15, SF-36, and CAT) by post.

Service utilisation data were collected from electronic patient records using a client service receipt inventory tool tailored for primary care services for 6 months prior to, and after, participation in the intervention. Healthcare costs were calculated according to NHS references/unit cost data information;^22^ this accounted for GP time commitments such as surgery consultations, telephone contacts, GP letters, and home visits.

### Care package elements

All participants received individual psychoeducation in respect of their MUS. This was delivered during baseline assessment (60–90 minutes) by a research assistant (psychiatry trainee doctor) with two main aims:

- to engage patients; and
- to extend and broaden their understanding of bodily functions. 

The psychoeducation included information about predisposing biological vulnerability, low pain thresholds, hyperarousal, and amplifying somatic styles of coping, perpetuating factors such as focused attention towards distressing bodily sensations (hypervigilance) and re-enforcement, and stress-tolerance models.

All﻿ patients were also offered a choice between two group interventions; this was done to allow patients to choose from an activity/movement-based intervention or one based on mindfulness. Both explicitly utilised body experiences.

#### Body-oriented psychological therapy

BOPT, offered under the name 'Strategies for Better Living Group (SBLG)', comprised 10 weekly sessions of 90 minutes each and was delivered by dance movement psychotherapists, who were trained to use a specific manual for BOPT and MUS. The intervention targets patients’ difficulties in acknowledging and expressing emotions, and aims to help them achieve fully embodied ways of relating to somatic symptoms. It identifies alternative behaviours in relation to coping with somatic symptoms, widens exploratory concepts and (bodily) self-images towards a more inclusive understanding of the inseparable nature of mental and physical processes, enriches and diversifies negative body images, activates resources (capabilities, bodily strength, and creativity), and sets the scene for (bodily, autonomic) self-regulation. The manual for the BOPT intervention and psychoeducation materials have been published together with a Template for Intervention Description and Replication checklist at (http://www.mus.elft.nhs.uk/Care-pathway/BOPT-MUS-manual-and-other-resources).

#### Mindfulness-based stress reduction

MBSR, comprising eight weekly sessions of 90 minutes each. This was delivered as an adapted version of the standard protocol^23 ^by a certified MBSR instructor. Written materials and audio CDs of guided meditations were provided to support home practice. MBSR therapy combines meditation, body-awareness techniques, and yoga exercises to enhance coping with distressing bodily symptoms such as pain. Techniques taught included: body scan; mindfulness of breath, body, feelings, thoughts, emotions; and mindful movement. The eight session themes were: introduction to MBSR, handling stress, and dealing with barriers; the power of being present; living all of your moments; learning about our stress reactions and how we deal with pain and other physical symptoms; coping with stress: using mindfulness to respond instead of react; thoughts are not facts; lifestyle choices — how can I best take care of myself?; and keeping your mindfulness alive.

## Statistical analysis

The authors used descriptive statistics, reported recruitment and retention figures for the study, and compared patients' initial and follow-up characteristics (somatic symptom levels, health-related subjective quality of life, and service utilisation). All values were expressed as mean and standard deviation (SD). Paired samples *t*-tests were conducted to analyse differences in outcome variables (calculation of 95% confidence intervals [CIs]; the significance level for hypothesis testing was set at 0.05). All statistical evaluations were performed with SPSS Statistics for Windows version 24.0.

## Results

**Figure 1:**
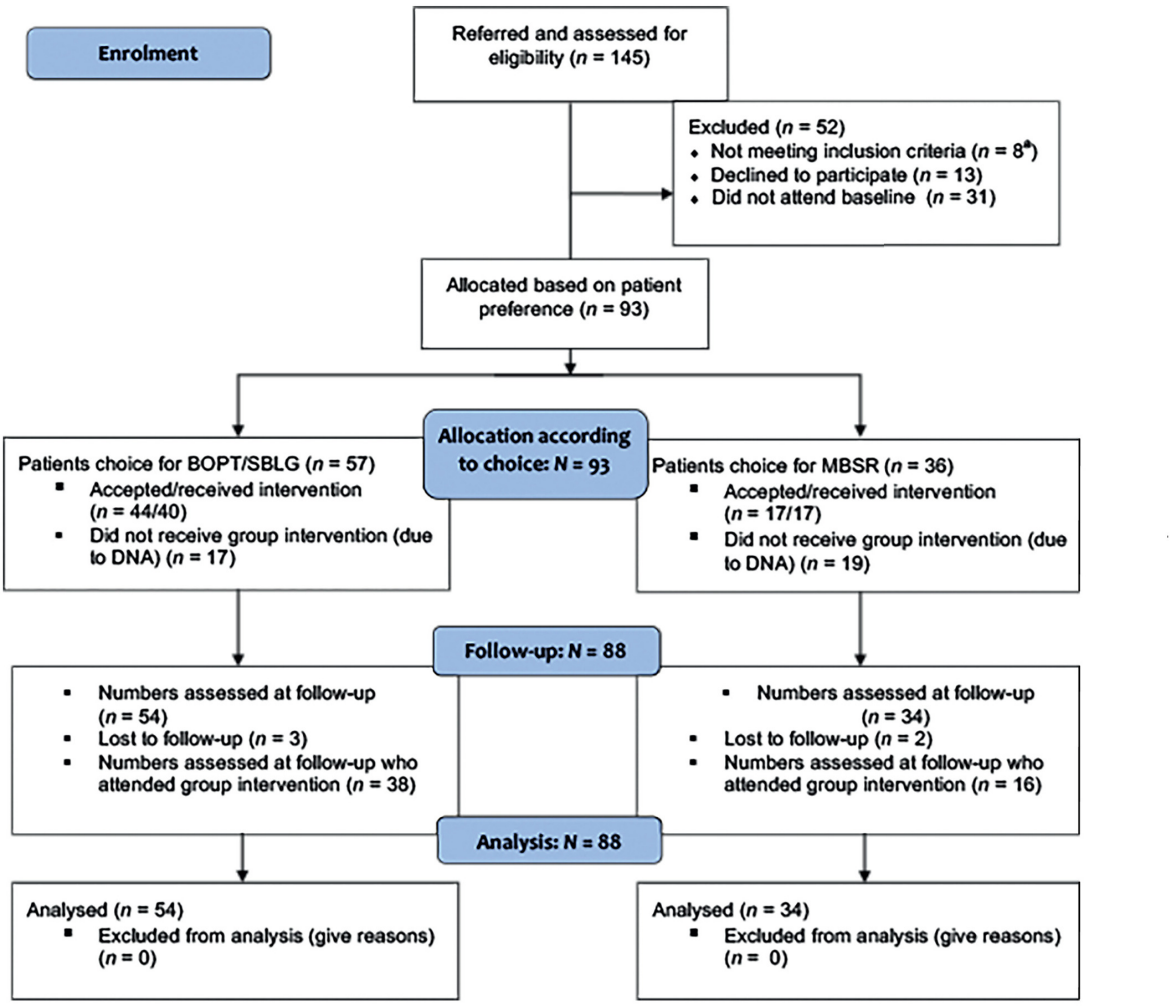
CONSORT flow diagram. ^a^1 patient aged <18 years with dementia, 1 patient out of area, 5 patients aged >75 years with high-level multimorbidity. BOPT = body oriented psychological therapy. DNA = did not attend. MBSR = mindfulness-based stress reduction. SBLG = Short Form for Strategies for Better Living Group.

Of the 145 referred patients, a total of 93 patients with a wide range of MUS conditions were included to participate and receive the care package. The study recruitment data and process is outlined in Figure 1.

### Participants' characteristics

Participant characteristics are shown in Table 1: 68 patients (73.1%) confirmed that they received family support in relation to their health problems; the mean number of hours was reported as 19 per week (range: 1–84 hours).

**Table 1.** Participants' characteristics.

	***n *(%)**
Mean age, years (range)	48 (21–75)
Sex	
Female	76 (81.7)
Male	17 (18.3)
**Ethinicity**	
White British	17 (18.3)
White other	7 (7.5)
Afro-Caribbean	6 (6.5)
Black African	10 (10.8)
Indian	11 (11.8)
Pakistani	16 (17.2)
Bangladeshi	23 (24.7)
Other	3 (3.2)
**Employment status**	
Unemployed	59 (63.4)
**Benefits received**	
State retirement pension	6
Statutory sick pay	9
Working Tax Credit	9
Housing Benefit	41
Council tax benefit	35
Disability Living Allowance mobility component	12
Disability Living Allowance care component	10
Incapacity benefit	5
Income support	20

PHQ-15 data on somatic symptoms was completed for all participants at baseline (mean total score 17.8, SD 5.7, indicating significant severity) and by 44 patients at follow-up. Baseline scores indicated high levels of somatic symptom severity: 74 patients rated the level of associated problems (an inability to do work, take care of things at home, or get along with other people) as 'extremely severe'. Most patients presented with multiple MUS, with the most frequently reported complaints being chronic/generalised aches and pains, headaches, back pain, nausea and fatigue; specific conditions such as irritable bowel syndrome and fibromyalgia were included. Comparative results are summarised in Table 2.

**Table 2.** Clinical baseline and follow-up comparison of somatic complaints and quality-of-life scores, using paired samples *t*-tests.

	***n***	**Mean**	**SD**	**Difference (95% CI)**	***P*-value**
**PHQ-15 total symptom score**					
Baseline	51	17.8	5.7		
Follow-up	51	14.2	6.8	3.79 (2.28 to 5.29)	<0.01
**SF-36 physical health component**					
Baseline	43	25.5	23.3		
Follow-up	43	33.5	35.9	–8.0 (–16.63 to 0.64)	0.69
**SF-36 mental health component**					
Baseline	42	35.8	25.5		
Follow-up	42	45.6	29.5	–9.8 (–18.44 to –1.18)	<0.05
**EQ-5D health score**					
Baseline	48	39.6	24.6		
Follow-up	48	47.4	25.8	–7.8 (–16.11 to 0.38)	0.61

PHQ-15 = 15-item Patient Health Questionnaire. SD = standard deviation. SF=36 = 36-item Short Form Survey.

### Interventions

For the group of 61 patients who accepted the invitation to participate in a group intervention (44 chose BOPT, 17 chose MBSR), the number of sessions attended varied between 1 and 10, with the mean being 2.4 sessions.

Comparing outcomes for those who attended a minimum of 5 sessions (*n* = 24) with those who had 1–4 sessions, better outcomes on all measures (symptom levels, quality-of-life scores, and service utilisation) were observed, but the results did not reach statistical significance. CAT scores obtained from 36 patients who participated in a group intervention demonstrated good satisfaction levels; mean scores across all questions were 6 or 7/10, while the question 'has treatment/care here been helpful for you?' had the best response with a mean rating of 8 or 9/10. The majority of these patients (77.0%) answered that they would want to attend more sessions if they were offered to them.

Themes from free-text comments regarding benefits of the interventions were identified as follows:

- shared understanding of problems with other patients;
- better coping with symptoms;
- learning new skills;
- feeling accepted with MUS problems;
- symptomatic relief; and
- empowerment and learning how to help oneself.

### Cost analysis

Healthcare expenditures were calculated from service utilisation data from GP electronic files; healthcare costs for the patients in the study sample over a period of 6 months prior to the baseline assessment were compared with those for the 6-month period after being enrolled in the study project. Non-healthcare costs associated with the health condition include social care costs, the secondary costs resulting from family or friends who provide support (even when not only and primarily as a result of the MUS health condition). The costs to the wider economy in terms of unemployment rates and state benefits paid were also considered. No changes in unemployment rates or access to state benefits were observed; neither did the hours of family or friends support reduce from baseline to follow-up. Table 3 lists the NHS unit costs as per standard documentation for all services that were included in the analysis; the mean reduction was £367 per patient over the 6 months after the intervention was carried out.

**Table 3.** Service utilisation and cost data comparison, using paired samples *t*-tests.

	***n***	**Mean**	**SD**	**Difference (95% CI)**	Unit cost, **£**	**Service cost calculation, £^^22^^**
**GP contacts**					39.72^a^	
Baseline	90	14.5	10.3			51 835
Follow-up	88	9.7	6.8	4.8 (2.88 to 6.77)^b^		33 905
**Specialist outpatient attendance contacts**					113	
Baseline	87	3.0	2.9			29 493
Follow-up	87	2.3	2.4	0.6 (0.03 to 1.19)^c^		22 611
**Hospital day case** **A&E visits **					140	
Baseline	87	0.9	1.5			10 962
Follow-up	87	0.4	0.8	0.5 (0.15 to 0.86)^c^		4872
**Physiotherapy sessions**					28	
Baseline	89	1.7	3.1			4236
Follow-up	88	0.4	1.2	1.3 (0.68 to 1.96)^b^		986
**Total cost, £**						
Baseline						96 526
Follow-up						62 374
Difference						34 152
Mean per participant						367
**Prescribed non-specific medication**						
Baseline	49	4.0	2.5			
Follow-up	49	4.1	3.8	–﻿0.04 (–0.92 to 0.84)^d^		
**Hours of support from family/friends**						
Baseline	33	16.2	20.9			
Follow-up	33	15.0	22.3	1.27 (–2.49 to 5.04)^d^		

A&E = accident and emergency. SD = standard deviation. ^a^Averaged cost per patient contact (lasting 11.7–17.2 minutes) or telephone consultation, prescription cost. ^b^ *P*<0.01. ^c^ *P*<0.05. ^d^Not significant.

The care pathway elements can all be delivered by senior psychological therapists, who have been trained to deliver the body-oriented treatments (BOPT and MBSR) and to conduct assessment/engagement and psychoeducation sessions. This can be also supported by sessional input from liaison psychiatrists if desired. Based on study figures, the authors calculated that the costs to run and administer the care pathway based on a full-time post (including salary, on-cost, office space, and equipment) would be approximately £57 000 per annum. At minimum/maximum capacity with 5–12 participants per group, this provides for 250 treated patients (allowing for a 35% drop-out rate from 400 referrals); the corresponding cost per patient is £228.

## Discussion

### Summary

This study explored the feasibility and evaluated outcomes of a novel primary care treatment package (a 'one-stop shop') for patients with MUS disorders, provided in one geographical cluster of GP surgeries in East London.

The results of this uncontrolled open study suggest that the majority of patients referred to the care pathway (approximately two-thirds) could be successfully engaged; this included a high percentage of patients from black and minority ethnic backgrounds.

The majority of participants chose the BOPT group (offered as 'Strategies for Better Living Group'). Those who received the care package reported significant reductions in self-reported symptom levels and corresponding improvements in their health-related subjective quality of life. Given the short-term nature of the intervention, significant changes of other social expenditures were not observed.

The two factors that may best explain the changes in this study are the body-oriented approach and the delivery of all components at primary care level. The care package utilised an approach that can be characterised as 'meeting the patients at home' — acknowledging that the nature and degree of their somatic complaints must be engaged with on a somatic level across all steps of the care pathway without challenging patients' explanatory beliefs. The results suggest that no single component of the care package (assessment/engagement, psychoeducation, or intervention) seems to account for the changes observed. Once engaged, patients benefited irrespective of the quantity of therapeutic inputs and the choice of interventions. Given the preliminary nature of the findings, this needs to be addressed in subsequent controlled efficacy studies.

### Strengths and limitations

In this study, emphasis was placed on GP engagement; the care pathway was developed and implemented in close collaboration with primary care colleagues and included a set-up phase providing practitioners with specific training, as well as raising awareness and fostering a better understanding of patients’ difficulties. It is particularly encouraging to see that the described symptom changes were reported for patients with high baseline scores, alongside improved subjective quality-of-life ratings and significant reductions in health service utilisation, as well as corresponding cost, indicating that the care package delivered clinical- and cost-effectivenes in parallel. When estimating the potential cost benefits of the care package, the cost associated with the delivery of the treatment elements has to be taken into account; this may vary from site to site according to available baseline resources.

This was not a controlled trial and different designs are required to detect treatment effects, but the results suggested clinically meaningful changes took place; patients were able to choose a group intervention based on their preference and in accordance with the main notion of the care pathway.

Data on symptom levels at follow-up could only be obtained for half of the sample, so results may be biased towards those who had better outcomes. Healthcare utilisation data, however, were collected from electronic records and, therefore, available for all participants.

### Comparison with existing literature

The findings are in line with previous studies that explored health benefits of BOPT for MUS,^24^ somatoform disorders,^16,25^ and specific 'psychosomatic' disorders.^26–28^

Evidence-based approaches at primary and secondary care level are few for patients with MUS.^8,10^ The relative paucity of randomised controlled trials for specific treatments is partially explained by heterogeneous patient characteristics and difficulties engaging them in any systematic form of psychological (talking) therapies.^11^ Data from a nationwide UK pathfinder project conducted by NHS England indicated low uptake and low attendance across 12 sites, with only one clinical site demonstrating benefits with symptom reductions, whereas in this study two-thirds of the 145 referred patients could be engaged to participate in the care pathway (University of Surrey Evaluation Team, unpublished data, 2015).

### Implications for research and practice

The existing evidence gap, along with the fact that most patients who experience persistent MUS are regarded as difficult to treat, indicates that research is required to address the efficacy of enhanced care models with mixed-method methodologies. Integrative and collaborative care pathway models that are firmly embedded within enhanced primary care practice seem to have advantages over traditional referral systems. The results of this uncontrolled open study suggest that the majority of patients referred to the care pathway (approximately two-thirds) could be successfully engaged; this included a high percentage of patients from black and minority ethnic backgrounds; this seems to be an advantage over traditional talking therapies. Psychological interventions for patients with somatisation problems should be delivered as intrinsic components of wellbeing strategies. Resource- and body-oriented approaches,^15,29^ as well as empathic support and symptom-immanent explanations,^30^ seem to be better accepted by patients with MUS than talking therapies, and more likely to promote better health outcomes.
